# PEPCK-M recoups tumor cell anabolic potential in a PKC-ζ-dependent manner

**DOI:** 10.1186/s40170-020-00236-3

**Published:** 2021-01-07

**Authors:** Petra Hyroššová, Marc Aragó, Juan Moreno-Felici, Xiarong Fu, Andrés Mendez-Lucas, Pablo M. García-Rovés, Shawn Burgess, Agnès Figueras, Francesc Viñals, Jose C. Perales

**Affiliations:** 1 Department of Physiological Sciences, School of Medicine, University of Barcelona, Feixa Llarga s/n, 08907 L’Hospitalet del Llobregat, Spain; 2 Center for Human Nutrition and Department of Pharmacology, University of Texas, Dallas, 75390 USA; 3 IDIBELL, Gran Via de l’Hospitalet 199, 08908 L’Hospitalet de Llobregat, Spain

**Keywords:** PEPCK, PEPCK-M, PCK2, Phosphoenolpyruvate carboxykinase, Cancer metabolism, PEP, Phosphoenolpyruvate, ER stress, Amino acid deprivation, PKC-ζ, ATF4, Activating transcription factor 4, AAR, Amino acid response, GCN2, TCA cycle, Cataplerosis, Serine/glycine metabolism, Proline metabolism, PYCR, PRODH/POX

## Abstract

**Background:**

Mitochondrial phosphoenolpyruvate carboxykinase (PEPCK-M; PCK2) is expressed in all cancer types examined and in neuroprogenitor cells. The gene is upregulated by amino acid limitation and ER-stress in an ATF4-dependent manner, and its activity modulates the PEP/Ca^2+^ signaling axis, providing clear arguments for a functional relationship with metabolic adaptations for cell survival. Despite its potential relevance to cancer metabolism, the mechanisms responsible for its pro-survival activity have not been completely elucidated.

**Methods:**

[U-^13^C]glutamine and [U-^13^C]glucose labeling of glycolytic and TCA cycle intermediates and their anabolic end-products was evaluated quantitatively using LC/MS and GC/MS in conditions of abundant glucose and glucose limitation in loss-of-function (shRNA) and gain-of-function (lentiviral constitutive overexpression) HeLa cervix carcinoma cell models. Cell viability was assessed in conjunction with various glucose concentrations and in xenografts in vivo.

**Results:**

PEPCK-M levels linearly correlated with [U-^13^C]glutamine label abundance in most glycolytic and TCA cycle intermediate pools under nutritional stress. In particular, serine, glycine, and proline metabolism, and the anabolic potential of the cell, were sensitive to PEPCK-M activity. Therefore, cell viability defects could be rescued by supplementing with an excess of those amino acids. PEPCK-M silenced or inhibited cells in the presence of abundant glucose showed limited growth secondary to TCA cycle blockade and increased ROS.

In limiting glucose conditions, downregulation of PKC-ζ tumor suppressor has been shown to enhance survival. Consistently, HeLa cells also sustained a survival advantage when PKC-ζ tumor suppressor was downregulated using shRNA, but this advantage was abolished in the absence of PEPCK-M, as its inhibition restores cell growth to control levels. The relationship between these two pathways is also highlighted by the anti-correlation observed between PEPCK-M and PKC-ζ protein levels in all clones tested, suggesting co-regulation in the absence of glucose. Finally, PEPCK-M loss negatively impacted on anchorage-independent colony formation and xenograft growth in vivo.

**Conclusions:**

All in all, our data suggest that PEPCK-M might participate in the mechanisms to regulate proteostasis in the anabolic and stalling phases of tumor growth. We provide molecular clues into the clinical relevance of PEPCK-M as a mechanism of evasion of cancer cells in conditions of nutrient stress.

**Supplementary Information:**

The online version contains supplementary material available at 10.1186/s40170-020-00236-3.

## Background

Phosphoenolpyruvate carboxykinase (PEPCK) (GTP; EC 4.1.1.32) catalyzes the GTP-dependent conversion of oxaloacetate (OAA) to phosphoenolpyruvate (PEP) from two enzymatically indistinct isozymes localized to the cytosol (PEPCK-C) or the mitochondria (PEPCK-M) [1, 2] and encoded by different nuclear genes (PCK1 and PCK2, respectively) [3]. Interestingly, both isozymes are differentially expressed and regulated. Whereas PEPCK-C is restricted to gluconeogenic and glyceroneogenic tissues (liver, small intestine, kidney cortex, and adipose tissue) and responds to insulin, glucagon, and dexamethasone, PEPCK-M is only stimulated by ER-stress effectors and it is widely expressed (i.e., T and B cells, pancreatic β cells, liver, neurons, and undifferentiated tissues such as embryonic stem cells and tumors). PEPCK-M effects on hepatic gluconeogenesis have been recently demonstrated [4], but its role remains elusive in non-gluconeogenic tissues such as tumor cells.

We identified PCK2 as a target gene for ATF4, the master regulator of ER and amino acid stress pathways. PEPCK-M was upregulated by effectors of this pathway by recruiting ATF4 to a consensus AARE site located at the PEPCK-M proximal promoter [5]. In these conditions, PEPCK-M activity is necessary to tip the balance towards cell survival. The importance of chronic ER stress in proper adaptive responses in cancer cells in vivo as part of their metabolic reprogramming hallmark for tumor progression bears weight to the significance of this pathway in tumor survival and progression.

Consistent with this view, we and others have demonstrated the relevance of PEPCK-M in cell growth and chemoresistance in several cancer cell models in vitro and in vivo [6–9]. However, the way PEPCK-M fluxes interact with tumor cell metabolism to promote survival are yet to be identified, and is the focus of the present work. On this, we have recently shown that PEPCK-M activity, and glucose availability, modulates cytosolic calcium signaling by upkeeping the pool of PEP, an inhibitor of SERCA, even in the presence of glucose in two models of colon carcinoma, HCT116 and SW480 [6]. Calcium then signals downstream effectors such as NFAT and c-Myc with implications on tumor cell biology. Thus, this regulatory axis is not only dependent on glucose availability but also on general nutritional status as PEPCK-M can flux carbons from several sources into the glycolytic pool.

We describe here the role of PEPCK-M in the preservation of the capacity for growth and survival in the cancer cell by ensuring the biosynthesis of amino acids such as proline, serine, and glycine. This pathway is important to offset glucose carbons with carbons from other sources such as glutamine or lactate, maintaining the TCA cycle, and in doing so, affect the general anabolic potential of the cell and its health status. Consistently, potentiation of resistance to glucose deprivation by the loss of PKC-ζ, a tumor suppressor [10], required PEPCK-M activity demonstrating that this enzyme serves an adaptative role in cancer metabolism, with significance in tumor growth, and further validates its potential as a therapeutic target.

## Methods

### Cell culture

Cervix (HeLa), and colon (HCT116 and SW480) carcinoma cell lines, and mouse *Kras*-V12 carcinogenic NIH-3T3 (NIH-3T3Kras) (a gift from Dr. Capella, IDIBELL, Spain) were cultured in DMEM supplemented with 10% FBS, 100 units/ml penicillin, 10 g/ml streptomycin, and 2 mM L-glutamine (all from Biological Industries, Israel) and incubated in a humidified atmosphere of 5% CO_2_ at 37 °C.

For experiments, unless stated otherwise, cells were grown in DMEM media with different glucose concentrations: 25 mM glucose (high glucose), 1 mM glucose (glucose exhaustion), and 0 mM glucose (glucose deprivation). Media were supplemented with 10% FBS, 100 units/ml penicillin, 10 g/ml streptomycin, and 2 mM or 4 mM (glucose deprivation) L-glutamine.

### RNA extraction and quantitative RT-PCR

Total RNA was extracted with the TRIsure^TM^ RNA isolation system (Bioline, Memphis, TN, USA) and transcribed with High Capacity cDNA Reverse Transcription Kit (Applied Biosystems, Foster City, CA, USA). QRT-PCR was performed using the TaqMan gene expression assay and 7900HT Real-Time RT-PCR system (Applied Biosystems, Carlsbad, CA, USA). Data were analyzed by the ∆∆Ct method to normalize with *TBP* and *GUSB* expression.

### Western blot

Cells were homogenized in RIPA buffer supplemented with protease and phosphatase inhibitors and centrifuged at 15,000 g for 15 min at 4 °C. Protein concentration was determined using the BCA protein assay kit (Thermo Scientific, Rockford, IL, USA), and equal amounts of protein (20–30 μg) were subjected to 8–12% SDS-PAGE and transferred to an Immobilon membrane (Millipore, Bedford, CA, USA). Blots were treated with primary antibodies, followed by the corresponding secondary antibody with horseradish peroxidase activity. Blots were developed using Pierce ECL reagent (Thermo Fisher Scientific, Waltham, MA, USA) in a Fujifilm LAS 3000 Intelligent Dark Box IV imaging system (Tokyo, Japan).

The following primary antibodies were used: anti-PEPCK-M (ab70359, Abcam, Cambridge, UK), anti-PEPCK-C (generous gift of Dr. Daryl K. Granner, Vanderbilt University, Nashville, TN, USA), anti-SOD2 (ab13534, Abcam), anti-p53 (ab26, Abcam), anti-p21 (sc397, Santa Cruz Biotechnology, Dallas, TX, USA), anti-gamma tubulin (T6557, Sigma-Aldrich, St. Louis, MO, USA), and anti-PKC-ζ (9372S, Cell Signaling Technology, Danver, MA, USA).

### Transduction

Protocols were performed as recommended by the manufacturer. For PCK2 knockdown, HeLa cells were infected with GIPZ Lentiviral TurboGFP shRNAs (Dharmaco, Lafayette, CO, USA; clone IDs: V3LMM_427490 and V3LHS_328126) and denominated sh1-PCK2 and sh2-PCK2, respectively. GIPZ non-targeting lentiviral TurboGFP shRNA (Dharmacon; clone ID RHS-4348) was used to produce negative control cells denominated shCtrl. After transduction, cells were selected with 1 μg/ml puromycin for 1 week. For overexpression of PCK2, HeLa cells were infected with a PCK2 Human ORFeome lentiviral particles (GeneCopoeia, Rockville, MD, USA; clone ID: LP-OL06695-LX304-0200-S) and denominated L-PCK2. Cells were selected with 2 μg/ml blasticidin for 1 week.

For PKC-ζ knockdown, HeLa and SW480 cells were infected with GIPZ Lentiviral TurboGFP shRNAs (Dharmacon; clone IDs: V3LHS_635000, V3LHS_372773, V3LHS_641464) and denominated shPKCζ #37, shPKCζ #63, and shPKCζ #64, respectively. The negative control, shPKCζ #Ctrl, was produced by infecting HeLa cells with GIPZ non-targeting lentiviral TurboGFP shRNA (Dharmacon; clone ID: RHS-4348). After transduction, cells were selected with 1 μg/ml puromycin for 1 week.

### Establishment of PCK2 knockout SW480 cell line with CRISPR/Cas 9 system

To generate a pool of SW480 cells lacking PCK2 (PCK2 CRISPR/Cas9 KO), guide RNAs (gRNA) designed to target PCK2 were synthesized, and annealed and cloned into the pSpCas9(BB)-2A-puro vector (Adgene, Watertown, MA, USA) as described previously [11], using an online gRNA design tool (CHOPCHOP; https://chopchop.cbu.uib.no). After 24 h post-transfection, puromycin was added for 24 h at 2 μg/ml for selection and subsequently single cells were selected in 96-well plates. The selected cells were tested for gene deletion by endonuclease assay and checked for protein knockdown by Western blot.

### PEPCK-M enzymatic activity

Cell extracts from confluent 150 cm^2^ tissue culture dishes were washed twice in PBS, trypsinized, and centrifuged at 150 g for 3 min at 4 °C. Cells were resuspended in 200 μl of ice-cold homogenization buffer (100 mM HEPES-NaOH pH 7.2, 0.1% triton™ X-100, 2.5 mM DTT) and lysed by performing 2 freeze/thaw cycles. Homogenates were cleared by centrifugation at 100000 g for 1 h at 4 °C. PEPCK-M activity was measured in the direction of phosphoenolpyruvate formation. Briefly, the reaction consisted of 100 mM of HEPES-NaOH (pH 7.2), 3 mM malic acid, 3 mM NAD, 2 mM MgCl_2_, 0.2 mM MnCl_2_, 37 mM DTT, 6 U/ml MDH, and the reaction was started by the addition of 0.2 mM of GTP. The amount of produced NADH is proportional to PEPCK activity. Reads were measured at 340 nm at 37 °C in a total volume of 1 ml using a DU® 800 spectrophotometer (Beckman Coulter, Brea, CA, USA).

### Immunohistochemistry

Tissue microarray panel (BCN962, Biomax, Rockville, MD, USA) containing multiple organ carcinoma and adjacent normal tissue was deparaffinized and rehydrated according to standard procedures. Antigen retrieval was performed by heating the slide in 10 mM sodium citrate buffer (pH 6) in a pressure cooker. The highest pressure was maintained for 3 min, and samples were let to cool down for 20 min. Endogenous peroxidase activity was inactivated by incubating samples in 6% H_2_O_2_ for 15 min.

Samples were blocked with 20% goat serum in PBS and then incubated ON with primary antibody against PEPCK-M (ab70359, Abcam) and peroxidase-based secondary anti-goat antibody. Antigen-antibody complexes were detected with a DAB peroxidase substrate kit (Dako Agilent, Santa Clara, CA, USA) according to the manufacturer’s protocol. Samples were counterstained with hematoxylin, dehydrated, and mounted with DPX. Fluorescent preparations were visualized, and images were captured with Nikon Eclipse 800 light microscope (Nikon, Tokyo, Japan).

### MTT assay

To assess cell viability, 0.5 mg/ml of MTT (M2128, Sigma-Aldrich, St. Louis, MO, USA) diluted in DMEM without phenol red was added to each well, and plates were incubated at 37 °C, 5% CO_2_ for 2 h. The formazan product was dissolved in isopropanol, and the absorbance of samples was measured using a microplate reader at a wavelength of 570 nm with background subtraction at 650 nm.

### Soft agar colony formation assay

Anchorage-independent growth was determined by plating 5000 cells in 1 ml of 0.35% agarose in 6 well plates. 0.7% agarose was mixed in a 1:1 ratio with DMEM media, supplemented with 10% FCS, 2 mM glutamine, and 1 mM or 25 mM glucose, respectively. A layer containing cells was overlaid on 0.5% agar in mQ water. Cells were fed with corresponding DMEM media and refed every 3–4 days. After 2 weeks, colonies were stained with MTT and counted.

### MitoSOX staining

MitoSOX™ Red mitochondrial superoxide indicator (M36008, ThermoFisher Scientific, Waltham, MA, USA) was used to measure the production of superoxide in mitochondria. Cells were treated with 5 μM MitoSOX™ Red in HBSS/Ca/Mg for 15 min at 37 °C and 5% CO_2_ protected from light. Cells were washed with HBSS/Ca/Mg. Subsequently, cells were trypsinized, resuspended in corresponding DMEM media without phenol red, and analyzed by flow cytometry with Gallios™ flow cytometer (Beckman Coulter).

### Metabolomics

Cells were plated in 6-well plates at 0.25 × 10^6^ cells/well and grown overnight in a growth medium. Next day, cells were washed with PBS and pre-treated with medium lacking glucose for 3 h and glucose-deprived medium supplemented with dialyzed FCS (dFCS) and 2 mM uniformly labeled [U-^13^C]glutamine was added for 4 h. In the case of high-glucose media (25 mM glucose), cells were pretreated with fresh complete DMEM media for 3 h and subsequently treated with DMEM media supplemented with dFCS and 2 mM [U-^13^C]glutamine for 4 h. At the end of cultivation, cells were washed twice with cold PBS, snap-frozen with liquid nitrogen, and harvested using an aqueous solution of MeOH (MeOH (80%)/H_2_O (20%)). The concentration of metabolites was analyzed by using mass spectrometer API 3200 triple quadrupole LC-MS/MS. Enrichment studies were analyzed by using GC/MS spectrometry (GC/MS Agilent 5975C). Proline enrichment and concentration of TCA cycle intermediates were analyzed in cells harvested in 1.8 ml of cold methanol/chloroform (2:1, v:v) and analyzed by using GC/MS spectrometry.

### Glucose assay

Glucose concentration in media was determined by colorimetry assay using a glucose oxidase and peroxidase method as recommended by manufacturer PGO (P7119, Sigma-Aldrich, Darmstadt, Germany). Absorbance was measured at 450 nm after 30 min of incubation at 37 °C.

### Xenograft models

5 × 10^6^ of SW480 WT or SW480 PCK2 CRISPR/Cas9 KO was injected in both flanks of female 5–6-week-old BALB/c nude mice (at least *n* = 3 per group). Similarly, 1 × 10^6^ of HeLa shCtrl, sh1-PCK2, or sh2-PCK2 was injected in two mammary fat pads of female 5–6-week-old BALB/c nude mice (at least *n* = 8 per group). Tumor volume was measured by calipering in two dimensions and calculated as [(short length × 2) × long length)]/2. After 15 days (SW480) or 23 days (HeLa), mice were euthanized by cervical dislocation, and the tumors collected for further analysis. All the animal studies were approved by the local committee for animal care (IDIBELL, DAAM 5766).

### Statistical analysis

Results are expressed as mean ± SEM. Statistical analysis was performed by one-way or two-way Anova (Sidak post hoc test) or unpaired two-tailed Student’s *t* test, using GraphPad Prism® software. Significance levels are one symbol = *p* < 0.05, two symbols = *p* < 0.01, and three symbols = *p* < 0.001.

## Results

**Fig. 1:**
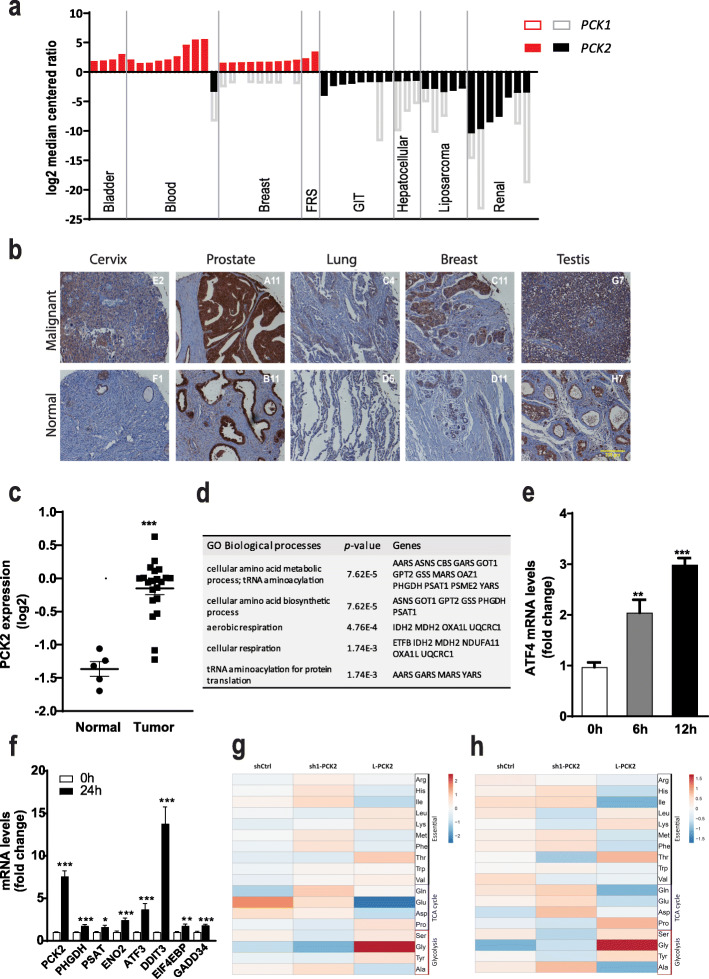
PEPCK-M is the PEPCK isoform preferentially expressed in tumors of diverse origin. **a** PCK1 and PCK2 expression in tumors from various tissues (FRS stands for female reproductive system, and GIT stands for gastro-intestinal). Graphical representation of log_2_ median-centered ratio of PCK1 (empty bars) and PCK2 (solid bars) present in each dataset analyzed using Oncomine at oncomine.org (analysis type: cancer versus normal, threshold at *P = 1E−5* and top gene rank 10%, *see references in Supplementary Table* *1*). Red or black colored boxes represent overexpressed or underexpressed for each dataset, respectively. Missing values for PCK1 expression were not significant or were not present in dataset. **b** Immunohistochemical analysis of PEPCK-M expression in selected types of tumors and their matched non-neoplastic tissue microarray BCN962 (US Biomax). Tissue was briefly counterstained with hematoxylin. Scale bar = 200 μm. **c** PCK2 gene expression (log_2_) from a microarray dataset comparing tumor samples of 35 patients with early stage cervical cancer and 5 normal cervical tissue samples. Statistical significance was determined by unpaired two-tailed Student’s *t* test. **d** Gene ontology analysis of 100 first hits co-expressing with PCK2 in human cervical cancer datasets analyzed using SEEK (Search-Based Exploration of Expression Compendium, Princeton), a gene co-expression search engine. *q* value minimum FDR and *p* value adjusted per multiple hypothesis testing Benjamini-Hockberg. **e**, **f** Quantitative real-time PCR analysis of mRNA levels of ATF4 at 6 h and 12 h (**e**), and selected genes at 24 h (**f**) in HeLa shCtrl cells grown in DMEM medium without glucose. Data is normalized to 0 h. Statistical significance compared with 0 h was determined by unpaired two-tailed Student’s *t* test. **g**, **h** Heatmap of amino acid distribution. Molar fraction of AA was determined in the cell extracts of HeLa cells grown for 24 h in high-glucose (25 mM) (**g**) and in glucose exhaustion (1 mM) (**h**) conditions. Data were processed and visualized by using ClustVistool with Pareto scaling (Metsalu & Vilo, 2015). Comparison of samples is realized within the rows

The expression of PEPCK-M in healthy organisms is mainly confined to the kidney, liver, pancreas, and small intestine. Yet, ONCOMINE dataset mining (Supplementary Table 1) demonstrates increased PCK2 mRNA levels upon malignant transformation in tissues where PEPCK-M is not originally expressed (Fig. 1a). Tumors originating from tissues where PEPCK-M is highly expressed in humans, such as the liver, pancreas, kidney, and gastrointestinal tract showed reduced expression. In contrast, PCK1 (PEPCK-C) expression was low in all types of tumors regardless of its levels in healthy tissue (Fig. 1a).

Immunohistochemical analysis of tissue microarrays with multiple normal and cancer tissue samples confirmed that the PEPCK-M signal was highly associated with malignancy, for example, in the prostate, lung, breast, testis, and cervix carcinomas (Fig. 1b). The healthy tissue from these organs (the glands of the prostate and uterus, and cells of seminiferous tubules) was minimally stained with PEPCK-M antibody. Finally, PEPCK-M mRNA levels were significantly elevated in clinical samples from cervical squamous cell carcinoma patients as compared to normal tissue (Fig. 1c; *P = 1.66E-5*), from either the metastatic or the non-metastatic dataset [12]. This points to a preferential role of the mitochondrial isoform of PEPCK in these cancers.

A gene ontology enrichment analysis of co-expressed genes in all available datasets of cervical cancer (Fig. 1d; *SEEK* at *seek.princeton.edu*) was then devised to expose PCK2 co-expressed pathways. Interestingly, gluconeogenesis was not enriched, and members of this pathway PCK1, FBP1, or G6PC1 are not co-expressors in this analysis. The hits on the enrichment table suggest a coordinated response with genes of anabolic potential, especially those related to amino acid metabolism and biosynthetic processes. Consistently, these genes are regulated by the integrated stress response (ISR) through ATF4 in a similar fashion to PCK2 [5], including ASNS, GPT2, SHMT1, PHGDH, or PSAT1. Induction of ER-stress in cervical carcinoma cells (HeLa) by glucose deprivation showed early upregulation of ATF4 (Fig. 1e) and other target genes of this stress response pathway such as DDIT3, GADD34, ATF3, and EIF4EBP1 at 24 h. As expected, PCK2 was also upregulated together with other genes, such as ENO2, PHGDH, or PSAT1 (Fig. 1f). All in all, this is suggesting a role for PEPCK-M within the general TCA cycle and amino acid biosynthesis nodes.

Thus, we examined the relative concentration (molar fraction) of amino acids in cell extracts of loss-of-function and gain-of-function HeLa squamous cell cervical carcinoma cells using mass spectroscopy (Fig. 1g, h). Amino acids with a relevant role in regulating the anabolic potential of the tumor, such as serine, glycine, proline, or threonine, showed increased molar fractions with increasing PEPCK-M expression with excess glucose in the media (Fig. 1g) and in a more physiological glucose exhaustion condition (Fig. 1h; 24 h after media refresh with 1 mM glucose which is depleted within the initial 12 h; *see* Supplementary Figure 2). Therefore, we focused our attention on understanding the contribution of PEPCK-M to the pathways involved in the synthesis and degradation of serine, glycine, and proline.

### PEPCK-M signature in tumors: serine/glycine metabolism

**Fig. 2:**
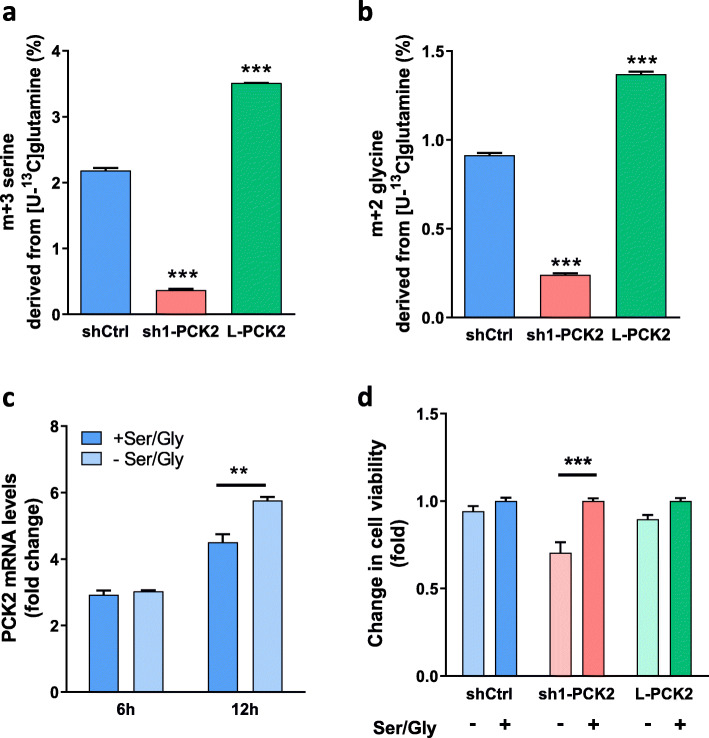
PEPCK-M is essential for Ser/Gly synthesis. **a**, **b** ^13^C enrichment of serine and glycine in HeLa cells exposed to 2 mM [U-^13^C]glutamine for 4 h in the DMEM media containing 10% dFCS and 0 mM glucose. Incorporation of ^13^C was analyzed using GC-MS. Statistical significance compared with shCtrl was determined by unpaired two-tailed Student’s *t* test. **c** Quantitative real-time PCR analysis of PCK2 mRNA expression levels in shCtrl HeLa cells grown in 0 mM glucose MEM media with or without 0.4 mM serine and 0.4 mM glycine (Ser/Gly) for 6 or 12 h. Values were normalized to time 0 h. Two-way ANOVA with Sidak multiple comparison post-test analysis indicate significance of Ser/Gly effect (*). **d** Cell viability was measured in HeLa cells using an MTT assay after 72 h of growth in 0 mM glucose MEM media with or without 0.4 mM serine and 0.4 mM glycine (Ser/Gly). The sensitivity of each cell line to the addition of serine and glycine is shown as fold change with respect to the viability in the presence of +Ser/Gly. Two-way ANOVA with Sidak multiple comparison post-test analysis indicate significance vs + Ser/Gly (*)

Direct incorporation of labeled carbons into serine and glycine from [U-^13^C]glutamine was readily observed in shCtrl cells, even in the presence of serine and glycine (DMEM media without glucose) (Fig. 2a). Silencing PEPCK-M as in sh1-PCK2 cells diminished the pool of fully labeled serine by 83%. Lower enrichment was also observed in other serine isotopologues (m + 1 was decreased by 82.7%, and m + 2 was 63.9% lower). Furthermore, overexpression of PEPCK-M significantly increased the pool of labeled species of serine by 1.6-fold (m + 3; Fig. 2a). Similarly, PEPCK-M-dependent distribution of label was present in glycine, which is synthesized from serine in a reaction catalyzed by serine hydroxymethyltransferase (SHMT) (Fig. 2b). The occurrence of fully labeled glycine in control cells was 0.9%, decreasing by 73.7% in PEPCK-M silenced cells (sh1-PCK2) and increasing 1.5 times when PEPCK-M was overexpressed (L-PCK2) (Fig. 2b).

Enrichment in serine and glycine correlated well with PEPCK-M activity present in silenced (sh1-PCK2 and sh2-PCK2) and overexpressed (L-PCK2) HeLa cells (PEPCK-M protein levels were 35% (sh1-PCK2), 44% (sh2-PCK2), and 250% (L-PCK2) of wild-type HeLa; Supplementary Figure 1). Also, neither the parental cell line nor any of the clones express the cytosolic isoform of PEPCK (PEPCK-C), and therefore changes in PEPCK-M expression do not translate to compensating upregulation of PEPCK-C (Supplementary Figure 1). Glutamine-labeled carbons were not measurable in the serine or glycine pools when excess glucose was present in the media (25 or 5 mM) regardless of the level of PEPCK-M activity (Supplementary Figure 3). Longer exposure of the cells to the label (24 h) was still insufficient to achieve the incorporation of glutamine carbon into serine or glycine in serine and glycine containing media (DMEM) and in the absence of exogenous serine and glycine (MEM) (Supplementary Figure 3).

The absence of serine and glycine (MEM media without glucose) increased PCK2 gene expression in shCtrl cells (Fig. 2c). In these conditions, PEPCK-M silenced cells (sh1-PCK2) were more sensitive to serine and glycine deprivation (Fig. 2d) as compared to shCtrl and PEPCK-M overexpressing cells. Similar results were obtained in HeLa wild-type cells using iPEPCK-2 [9], a potent PEPCK-M inhibitor (Supplementary Figure 4).

### PEPCK-M signature in tumors: proline metabolism

**Fig. 3:**
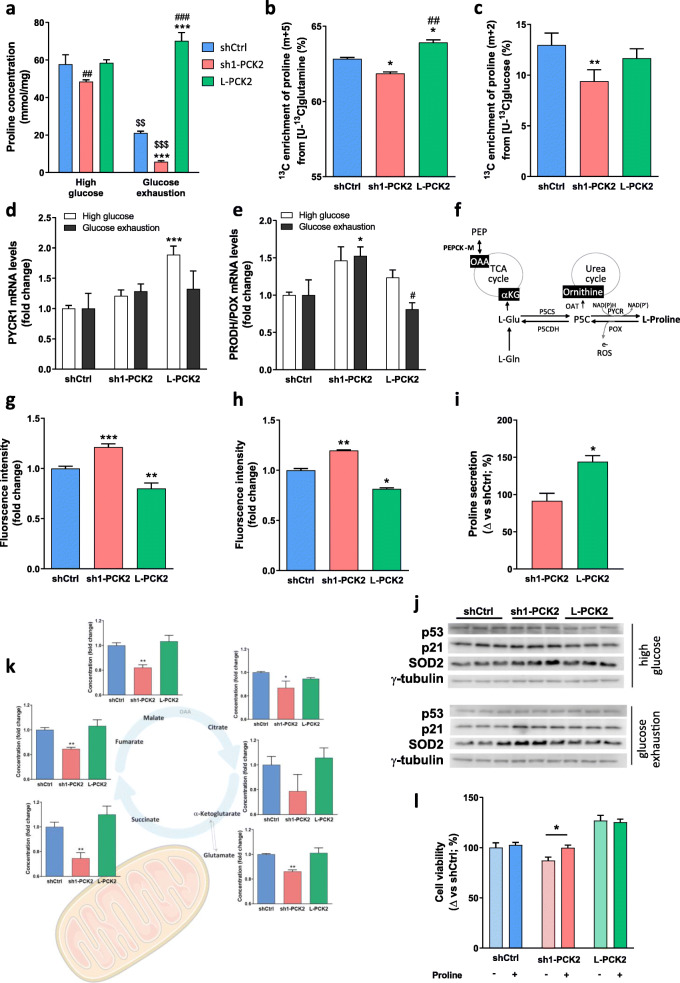
PEPCK-M influences proline metabolism. **a** Concentration of proline in HeLa cells grown for 24 h in high-glucose (25 mM) and glucose exhaustion (1 mM) conditions. Amount of proline was normalized to mg of protein. Concentration was analyzed by LC-MS. Two-way ANOVA with Sidak multiple comparison post-test analysis indicate significance versus shCtrl within media treatment (*), significance between sh1-PCK2 and L-PCK2 within media treatments (#), and significance of media treatment effect ($). **b** Full ^13^C enrichment of proline in HeLa cells exposed to 2 mM [U-^13^C]glutamine for 4 h in the DMEM media containing 10% dFCS and 0 mM glucose. Full incorporation of ^13^C was analyzed using GC-MS (+4 carbon in GC-MS analysis corresponding to m + 5 on LCMS). One-way ANOVA with Sidak multiple comparison post-test analysis indicate significance versus shCtrl (*), and significance between sh1-PCK2 and L-PCK2 (#). **c** ^13^C enrichment of proline in HeLa cells was exposed to 25 mM [U-^13^C]glucose for 4 h in the DMEM media containing 10% dFCS. Incorporation of ^13^C was determined by the +2 carbon on GC-MS and corresponded to m + 2 on LCMS. One-way ANOVA with Sidak multiple comparison post-test analysis indicate significance versus shCtrl (*), and significance between sh1-PCK2 and L-PCK2 (#). **d**, **e** Quantitative real-time PCR analysis of *PYCR1* (**d**) and *PRODH/POX* (**e**) mRNA expression levels in HeLa cells grown for 24 h in high-glucose (25 mM) and glucose exhaustion (1 mM) conditions. Values were normalized to shCtrl for each condition. One-way ANOVA with Sidak multiple comparison post-test analysis indicate significance versus shCtrl (*), and significance between sh1-PCK2 and L-PCK2 (#). **f** Proline metabolism pathway and its implications for ROS formation. **g**, **h** Production of mitochondrial superoxide in HeLa cells treated for 3 h in glucose exhaustion (1 mM) (**g**) and high-glucose (25 mM) (**h**) conditions. Superoxide was quantified using MitoSOX fluorescent marker, and mean intensity was measured by flow cytometry. Statistical significance compared with shCtrl was determined by unpaired two-tailed Student’s *t* test. **i** Secretion of proline in high-glucose DMEM media (not containing proline) by HeLa cells after 24 h. Concentration of proline was normalized to protein and plotted as % of shCtrl secretion. Concentration was analyzed by LC-MS. Statistical significance between sh1-PCK2 and L-PCK2 was determined by using unpaired two-tailed Student’s *t* test. **j** Western blot analysis of p53, p21, and SOD2 in HeLa cells grown for 24 h in high-glucose (25 mM) and glucose exhaustion (1 mM) conditions. Gamma tubulin was used as loading control. **k** Concentration of TCA cycle intermediates in HeLa cells grown 24 h in high-glucose media (25 mM). Concentration of metabolites was normalized to protein, and fold change was calculated to shCtrl cells. Concentration was analyzed by GC-MS. One-way ANOVA with Sidak multiple comparison post-test analysis indicate significance versus shCtrl. **l** Cell viability was measured in HeLa cells using an MTT assay after 48 h growth in DMEM media lacking glucose with or without supplementation with proline (5 mM). Data are normalized to shCtrl cells grown in the absence of proline. Statistical significance was determined by using unpaired two-tailed Student’s *t* test

Next, we measured proline concentration in cell extracts in the absence of exogenous proline (DMEM). Lower levels of proline were observed in sh1-PCK2 cells (48.4 ± 1.02 mmol/mg) when compared to shCtrl (57.78 ± 5.02 mmol/mg) and L-PCK2 (58.46 ± 1.69 mmol/mg) (Fig. 3a). More marked differences, that tightly correlated with PEPCK-M activity, were observed under glucose exhaustion; with overexpressing PEPCK-M cells showing highest (70.25 ± 4.33 mmol/mg) proline levels as compared to shCtrl (21.13 ± 0.93 mmol/mg) or sh1-PCK2 cells (5.79 ± 0.56 mmol/mg).

Furthermore, L-PCK2 cells showed increased enrichment of ^13^C carbons from [U-^13^C]glutamine into proline (m + 5) as compared to reduced labels measured on the sh1-PCK2 group (Fig. 3b), correlating with PEPCK-M levels. In the presence of excess glucose though, fully labeled proline was equally enriched in all the clones (Supplementary Figure 5B). In these conditions, we wanted to examine whether the synthesis of proline from glucose was being affected by the activity of PEPCK-M measuring [U-^13^C]glucose (25 mM) incorporation. PEPCK-M silencing showed a small but significant impact on the incorporation of carbons from glucose into the proline (m + 2) pool (Fig. 3c), consistent with our cell extract proline measurements. All in all, these data suggest an indirect role for PEPCK-M on proline metabolism that depends on the metabolic status of the cell.

Reduced proline content in shCtrl and sh1-PCK2 cells going from excess glucose to glucose exhaustion conditions suggest increased proline degradation. Proline can be metabolized to glutamate to feed the TCA cycle and the respiratory chain. Difference in proline dehydrogenase (PRODH/POX) expression between high-glucose and glucose exhaustion condition was significantly higher in sh1-PCK2 cells (Supplementary Figure 5A), suggesting increased proline metabolism under this condition. Interestingly, POX and PYCR1 mRNA were unresponsive in cells overexpressing PCK2 (L-PCK2) when compared high-glucose to a glucose exhaustion media (Supplementary Figure 5A), suggestive of a certain degree of resistance to the signals that activate this proline metabolizing pathway. Furthermore, PYCR1 was significantly increased in the presence of excess glucose exclusively in L-PCK2 cells, an indication of enhanced anabolism in these cells (Fig. 3d).

Consistent with higher utilization of proline, PRODH/POX mRNA levels showed increased expression in sh1-PCK2 cells both in high-glucose and glucose exhaustion conditions (Fig. 3e) when compared to shCtrl or L-PCK2 cells. ROS is produced during proline degradation by PRODH/POX as an electron is passed to the electron transport chain (ETC) (Fig. 3f) [13–15]. Indeed, sh1-PCK2 showed increased ROS in glucose-exhaustion (Fig. 3g) and glucose-abundant (Fig. 3h) conditions, as quantified using a mitochondrial ROS indicator (MitoSOX). Interestingly, the L-PCK2 clone demonstrated reduced ROS production in glucose exhaustion conditions beyond shCtrl, in parallel with higher net secretion of proline into the media (Fig. 3i), further indicating a link between proline metabolism and ROS homeostasis that is closely associated to PEPCK-M activity in the tumor cell. In effect, silencing PEPCK-M concurred with enhanced ROS-mediated signaling as evidenced by increased SOD2 and TP53/P21 proteins in sh1-PCK2, both in the presence of abundant glucose in the media (Fig. 3j; *top panel*) and in glucose exhaustion conditions (Fig. 3j; *bottom panel*). Also, alterations coincided with a systematic block on the TCA cycle as the pool of most intermediates was significantly lower in sh1-PCK2 cells (Fig. 3k).

As PEPCK-M silencing (sh1-PCK2) alters proline metabolism and ROS homeostasis, we aimed to assess whether these stimuli reduced cell viability. Indeed, viability was reduced in PCK2-silenced cells and restored at least in part by proline supplementation in the media (Fig. 3l). This was also observed in a different model, HCT116 colon carcinoma, after inhibition of PEPCK-M using iPEPCK-2 (Supplementary Figure 5C).

### PKC-ζ downregulation growth advantage requires PEPCK-M activity

**Fig. 4:**
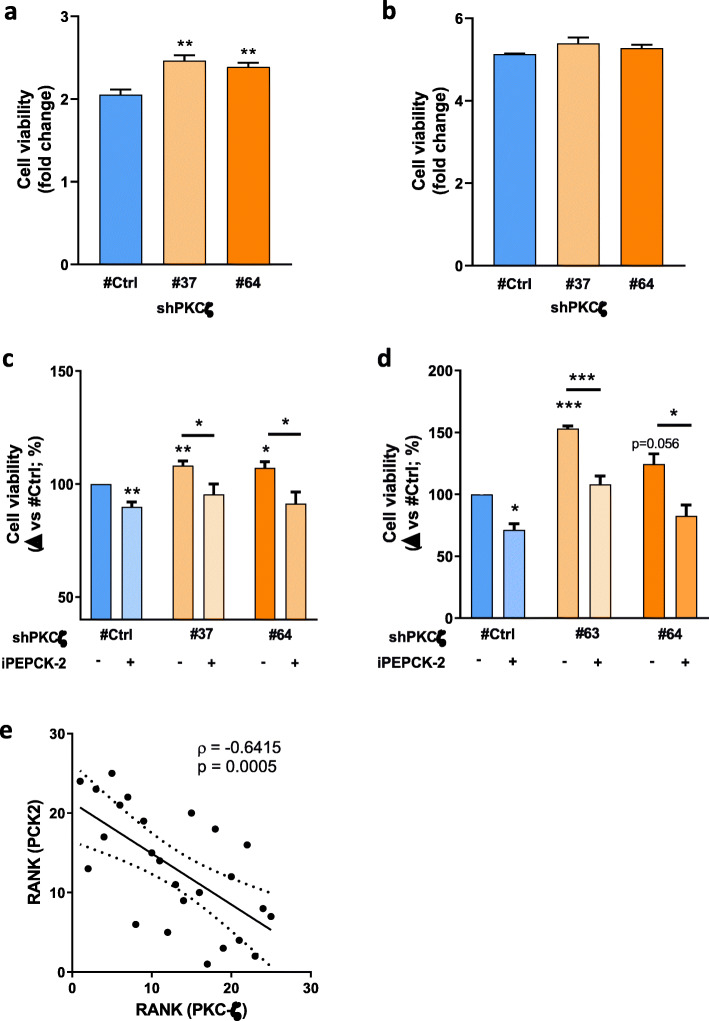
Resistance to glucose deprivation enhanced by PKC-ζ requires PEPCK-M. **a**, **b** Effect of PKC-ζ silencing on cell viability. MTT cell viability assay was measured in HeLa cells growing 72 h in high-glucose media (**a**) and glucose exhaustion media (**b**). Fold change was calculated to values measured at 0 h for each cell line separately. Statistical significance compared with shCtrl was determined by unpaired two-tailed Student’s *t* test. **c**, **d** MTT cell viability assay of control and PKC-ζ silenced HeLa (**c**) and SW480 (**d**) cells treated with inhibitor of PEPCK-M (iPEPCK-2; 5 μM) or vehicle (DMSO). Cells were grown for 72 h in media lacking glucose. Data was normalized to shPKCζ #Ctrl with DMSO. Statistical significance compared with shCtrl was determined by unpaired two-tailed Student’s *t* test. **e** Spearman’s rank order correlation analysis between the protein expression levels of PKC-ζ and PEPCK-M in HeLa sh1-PCK2 cells treated with shRNAs against PKC-ζ. Expression levels were quantified in 24 different clones by densitometry of immunoblots

Several tumor survival pathways are involved in the recruitment of alternative metabolic pathways, including the downregulation of the PKC-ζ tumor suppressor [10], that triggers the utilization of glutamine carbon when glucose is limiting to cope with amino acid synthesis for survival and growth. We confirmed that cell survival is boosted after PKC-ζ downregulation in cervix (HeLa) carcinoma cells in glucose exhaustion conditions (Fig. 4a), but not in conditions of excess glucose (Fig. 4b), using two independent clones of HeLa cells constitutively expressing shRNA against PKC-ζ. We then examined whether the advantage observed in cell growth upon PKC-ζ downregulation required the PEPCK-M axis, as it is the only pathway that connects TCA carbons with routes of amino acid synthesis, as presented above. Treatment with iPEPCK-2 to block PEPCK-M activity blunted the growth advantage provided by the downregulation of PKC-ζ (Fig. 4c). This observation was confirmed in a different model, SW480 colon carcinoma, where downregulation of PKC-ζ provided a survival advantage in the absence of glucose that was again lessened when PEPCK-M was inhibited using iPEPCK-2 (Fig. 4d). Further, we observed strong anti-correlation fit between PKC-ζ and PEPCK-M (Fig. 4e) in HeLa sh1-PCK2 cells treated with shRNAs against PKC-ζ, altogether suggesting cross-regulation of both pathways in conditions of glucose limitation.

### The implication of PEPCK-M in tumor growth in patho-physiological models

**Fig. 5:**
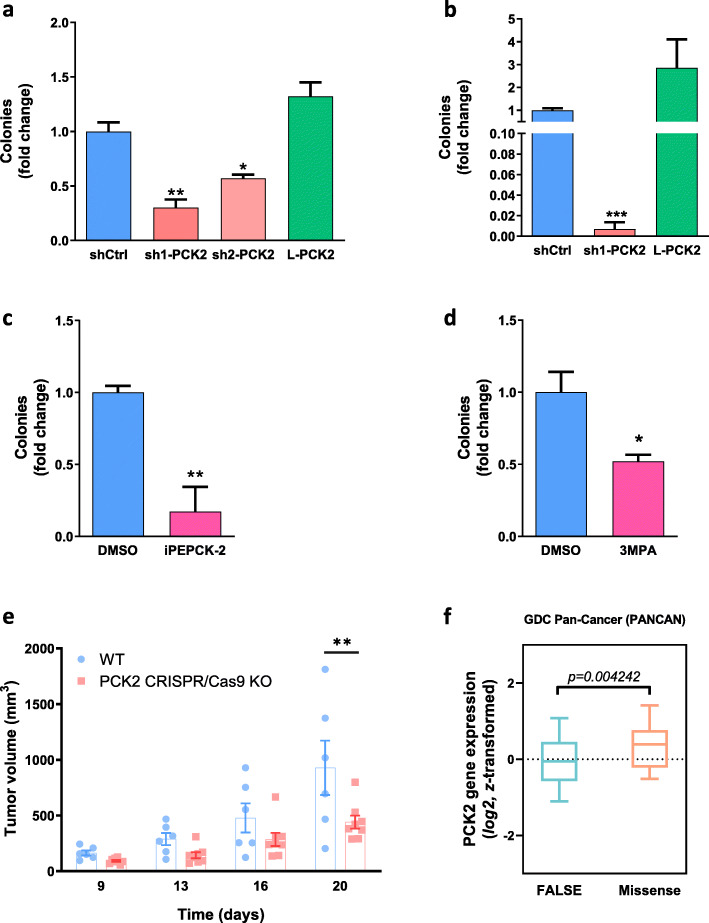
Effects of PEPCK-M activity change on anchorage-independent and xenograft growth. **a–d** Quantification of anchorage independent growth of HeLa and NIH-3T3Kras cells. Cells were seeded in semisolid agarose prepared with DMEM media 1:1 and grown for 4 weeks. At the end of incubation, cells were stained with MTT and number of spheres was counted. HeLa cells were grown in the presence of high-glucose media (25 mM) (**a**, **c**) or glucose exhaustion media (1 mM) (**b**). During the whole experiment, HeLa shCtrl cells in **c** were treated with PEPCK-M inhibitor (iPEPCK-2; 5 μM) or vehicle (DMSO). NIH-3T3Kras cells in **d** were grown in high-glucose media and treated with PEPCK-M inhibitor (3MPA; 100 μM) or vehicle (DMSO). Treatment media was refreshed every 3–4 days. Results are presented as fold change to shCtrl or DMSO, respectively. Statistical significance to shCtrl or DMSO was determined by unpaired two-tailed Student’s *t* test. **e** Tumor growth of SW480 WT and PCK2 CRISPR/Cas9 KO cells implanted into the flanks of BALB/C nude mice. Significance was determined using two-way ANOVA with Sidak multiple comparison post-test analysis. **f** Gene expression comparison analysis of tumor samples in the GDC Pan-Can dataset. Tumors presenting missense (Missense; *n* = 48) variant mutations are compared to non-variant tumors (FALSE; *n* = 11542). Unpaired *t* test analysis with Welch’s correction was used to determine statistical significance

The ability to form spheres in an anchorage-independent manner (in semisolid media without attachment) is an indicator of tumorigenic and metastatic potential [16, 17], as it mimics diminished nutrient availability and increased microenvironment stress in the tumor. HeLa cells grown in soft agar were able to form spheres after four weeks of incubation (Fig. 5a; shCtrl), and sphere formation was reduced severely in PEPCK-M silenced cells (by 70% in sh1-PCK2 and by 43% in sh2-PCK2, when compared to shCtrl) (Fig. 5a). Similarly, sphere formation was reduced in PEPCK-M-silenced cells cultured continuously at 1 mM glucose in the media (Fig. 5b). Overexpression of PEPCK-M in HeLa cells (L-PCK2) slightly increased the number of spheres; however, this increase was not significant.

These results were confirmed in other models using a pharmacological approach (Fig. 5c, d). Thus, HeLa wild-type cells or a cell line with constitutive expression of a mutant *Kras* in transformed mouse fibroblasts (NIH-3T3Kras cells) treated with iPEPCK-2 or 3MPA, reduced sphere formation ~ 83% (Fig. 5c) and ~ 48% (Fig. 5d), respectively. Interestingly, mass cultures of shRNA-silenced PEPCK-M cells described here (sh1-PCK2 and *s*h2-PCK2) and grown as fat pad grafts in nude mice for several weeks, reverted to wild-type, shCtrl levels of PEPCK-M expression suggesting significant selective pressure in the tumor to favor cells with higher levels of PEPCK-M (Supplementary Figure 6). Therefore, we designed null mutants (PCK2 CRISPR/Cas9 KO) to further investigate the capacity of PEPCK-M to sustain tumor growth in vivo in a xenograft model*.* A colon carcinoma model, SW480 was selected instead of HeLa because it carries only one copy of chromosome 14 where the PCK2 gene is located, as compared to the multiple copies found in the HeLa genome, simplifying CRISPR/Cas9 use. These models have been previously characterized and show similar effects on cellular viability as the HeLa model of PEPCK-M knockdown presented here [9]. Both PCK2 CRISPR/Cas9 KO and wild-type (WT) cells were then injected into the flanks of immunocompromised NOD/SCID mice and allow to grow in the absence of external nutritional stress (chow). Tumor growth was blunted in PCK2 CRISPR/Cas9 KO cells as compared to WT (Fig. 5e).

## Discussion

Metabolic reprogramming is a hallmark of cancer that helps tumors cope with anabolism and energy demands for growth and invasion [18]. In this article, we propose PEPCK-M as a key player in this hallmark when glucose levels drop and tumors must shift to use other substrates, such as glutamine. An expression signature for mitochondrial PEPCK (PEPCK-M) is present in most cancer cells examined. On the contrary, most tumors analyzed showed very low or no expression of PEPCK-C, the cytosolic form of this enzyme. Interestingly, the tumors originating from tissues that originally express cytosolic and mitochondrial isoforms, such as the kidney and liver, maintain expression of both upon malignancy. However, PEPCK-C expression levels dropped in these tumors, as reported elsewhere in hepatocellular carcinoma and colon cancer [19, 20]. PEPCK-M expression, on the other hand, did not decrease as much in the tumors originating from the liver. These data point to a deleterious impact of gluconeogenic pathways in cancer progression reinforced by similar behavior for glucose-6 phosphatase and fructose bisphospatase-1 in human malignancies of gluconeogenic tissues [20, 21]. PEPCK-C might participate in specific phases of the tumor progression in adaptative responses that are key to cancer development as it is suggested by its participation in some metabolic adaptations in hepatocarcinoma [22] and melanoma [23].

A clue on the mechanism for the integrative role of PEPCK-M in cancer metabolism arises from its transcription regulation by ATF4 under nutrient stress [24, 25]. In these conditions, we demonstrate the functional relevance of the pathway to feed ^13^C-labeled carbons from glutamine into phosphoenolpyruvate (PEP), serine, and glycine, demonstrating that cataplerosis of TCA cycle 4-carbon intermediates towards PEP, serine, and glycine was specifically dependent on PEPCK-M flux. Even though enrichment was seemingly low, labeling incubation was very short by design as we aimed to avoid saturation of the labeling pattern in various TCA cycle intermediates to properly assess cataplerosis. It is also important to mention that labeling studies were performed in the presence of exogenous serine and glycine. This may lead to an underestimation of isotope labeling due to equilibration with unlabeled exogenous sources as pointed out by DeNicola et al. [26]. The nearly linear relationship with PEPCK-M protein in silenced (sh1-PCK2; ~ 35%), wild-type (shCtrl; 100%), and overexpressed (L-PCK2; ~250%) clones suggests a good control of the flux rates towards this pathway exerted by PEPCK-M, unlike the low control coefficient shown for PEPCK-C in liver gluconeogenesis [27]. These data are consistent with the observation from Vincent et al. that carbons originating from glutamine flux fed into serine under glucose deprivation via PEPCK-M [28] in A549 NSCLC cells, known to withstand glucose deprivation and even proliferate in the presence of glutamine. In contrast, HeLa cells did not cope well with the severe stress-induced by total glucose depletion as they stop to grow and total cell number was decreased over time, although PEPCK-M overexpression did enhance somewhat the ability of these cells to survive in these conditions.

PEPCK-M overexpressing cells showed an anabolic phenotype, with higher serine consumption from the media (data not shown), and a higher molar fraction of amino acids synthesized from either PEP (tyrosine), 3PG (serine and glycine), or intermediaries of the TCA cycle (aspartate and proline). Besides, proline secretion was significantly increased in HeLa cells with overexpressed PEPCK-M which correlated with higher expression of PYCR enzyme that converts pyrroline-5-carboxylate to proline, in both glucose concentrations tested. Indeed, lower proline concentration in PEPCK-M silenced cells, together with higher expression of POX, an enzyme involved in the catabolism of proline, points to increased proline turnover or consumption in PEPCK-M limiting conditions, and supports a role for PEPCK-M in the biosynthesis of proline, albeit indirectly.

The importance of proline for cancer growth might also involve the NADPH/NADP+ equilibrium and the generation of ROS consequence of proline cycling [29, 30]. Our data is consistent with this view, and with previous observations that increased proline metabolism correlated with higher ROS production at the PRODH/POX node, and a blockade in TCA cycle flux [15], a general mechanism to cope with hypoxic stress [31]. These events identify a metabolic disturbance that bear on the health status of the cells with reduced PEPCK-M activity (i.e., sh1-PCK2), albeit with minimal effects on cell viability and apoptosis in the absence of nutrient stress [5, 6, 9].

Based on the results shown, and previous data on neuronal progenitors suggesting a role for PEPCK-M in ECM [32], we believe that this pathway might be relevant to the proteostatic phenotype. In this regard, its role in the control of the anabolic potential of the cell is very consistent with the observation that PEPCK-M loss has a greater impact on 3D culture homeostasis (i.e., anchorage-independent colony formation) than what might be inferred from cell culture silencing studies [6, 7, 9]. Moreover, the synthesis of proline is activated by c-Myc [14], and PEPCK-M might influence this pathway through its regulation of calcium fluxes by modulating PEP levels and SERCA activity in cancer cells [6, 33], that in turn triggers the CAMKIIγ-dependent phosphorylation and stabilization of c-Myc. Interestingly, PEPCK-M can impact on metabolism by regulating the PEP levels even in the presence of excess glucose in HCT116 cells [6]. However, in the present study using HeLa cells, we have not been able to measure specific flux of carbons from glutamine into serine and glycine in these conditions, even with extended incubation times. An indirect contribution of PEPCK-M to the glycolytic flux cannot reconcile these data since glycolytic flux measured in HCT116 and SW480 cells with [5-^3^H]-glucose showed no changes after PEPCK-M inhibition [6]. A similar conundrum was identified by Maddocks et al. who detected the formation of unlabeled serine in HCT116 cells grown in the presence of fully labeled glucose and the absence of exogenous serine and glycine [34], pointing to the implication of a different pathway towards serine synthesis, which might involve PEPCK-M. This idea is also supported by the phenomenon occurring in adipocytes, where glyceroneogenesis is the main source of triglycerides even in the presence of glucose [35].

In addition to c-Myc, other central regulators of metabolism in the tumor have recently surfaced that might directly impact on the PEPCK-M pathway. One such example is PKC-ζ, a member of the atypical PKC family whose loss has been shown to enhance glutamine utilization and serine and glycine synthesis in conditions of glucose deprivation [10]. The role of PKC-ζ in glucose limiting conditions was corroborated in our lab both in the HeLa and the SW480 cell models using shRNA. In these models, PEPCK-M was necessary to support the successful metabolic reprogramming of PKC-ζ loss. Furthermore, regulatory anti-correlation between PEPCK-M and PKC-ζ levels were observed in several independent clones tested where PKC-ζ expression was silenced using various sequences of shRNA, pointing towards the interaction of both pathways in response to nutrient stress. The relevance of these findings for tumor biology is highlighted by an initial analysis of the expression of PCK2 in tumors from the GDC Pan-Can dataset showing increased expression of the gene in tumors presenting missense variant mutations as compared to non-variant samples (Fig. 5f). This data, together with the abundant information on the importance of PKC-ζ suppression in tumor growth in vivo [36, 37], support a relevant link between these two pathways in connection with tumor viability and growth.

## Conclusions

All in all, our work described mechanisms for metabolic flexibility in cancer cells that are dependent on PEPCK-M activity. PEPCK-M-driven cataplerosis, in sync with PKC-ζ suppression, adds a dynamic provision of substrates to cancer cells for keeping-up with stress and promote anabolism.

The mechanism for the supportive role of PEPCK-M in cancer cells opens a therapeutic window and enhances our understanding of cancer metabolism. Finally, these studies support this enzyme as a target in this pathology and will serve as a set point for continuing our development of new therapeutic strategies involving related pathways.

## Supplementary Information

**Additional file 1: Supplementary Table 1.** Oncomine datasets reference list. Log2 median centered ratio of PCK1 and PCK2 in different cancers analyzed with Oncomine (https://www.oncomine.org; Rhodes et al., 2004). Analysis type: Cancer versus Normal, threshold at *p*=1E-5 and top gene rank 10%. Missing values for PCK1 expression were not significant or were not analyzed in given dataset. Reference to original study is listed.

**Additional file 2: Supplementary Figure 1.** HeLa model with stable silencing and overexpression of PEPCK-M. Silencing and overexpression were obtained using lentiviral vectors containing inserts of shRNA against PCK2 or inserts of PCK2 cDNA, respectively. **(A)** Western blot analysis of mitochondrial and cytosolic PEPCK expression levels in HeLa cells with altered PEPCK-M expression levels: overexpressed (L-PCK2), basal (shCtrl and WT) and knocked down PEPCK-M (sh1-PCK2 and sh2-PCK2). As a positive control of PEPCK-C expression, extracts from mouse liver and Fao hepatoma cells were used. **(B)** Western blot quantification of PEPCK-M protein abundance in HeLa modified lines. PEPCK-M expression was normalized by gamma tubulin. Results are represented as fold change to HeLa WT. One-way Anova with Sidak multiple comparison post-test analysis indicate significance versus WT*.* **(C)** PEPCK-M enzymatic activity in HeLa shCtrl, sh1-PCK2, sh2-PCK2 and L-PCK2 cells grown in basal conditions was measured by production of NADH. One-way Anova with Sidak multiple comparison post-test analysis indicate significance versus shCtrl. **Supplementary Figure 2.** Time course of glucose depletion under glucose exhaustion conditions. HeLa shCtrl cells were washed 3 times with PBS and treated with DMEM medium containing 1 mM glucose. Concentration of glucose in medium was measured every 3 h, up to 24 h. **Supplementary Figure 3.** ^13^C enrichment of serine and glycine in HeLa cells. **(A)** Cells were exposed to 2 mM [U-^13^C]glutamine for 4 h in the DMEM media containing 10% dFCS and 25 mM glucose. Incorporation of ^13^C into proline was analyzed using GC-MS. **(B)** HeLa shCtrl cells were exposed to 2 mM [U-^13^C]glutamine for 4 h in the DMEM media containing 10% dFCS and 25 mM glucose or media lacking serine and glycine (MEM) containing 10% dFCS and 5 mM glucose. Incorporation of ^13^C was analyzed using GC-MS. Negative values were set as 0. **Supplementary Figure 4.** PEPCK-M inhibition with iPEPCK-2 effects on viability are dependent on Ser/Gly. MTT cell viability assay of HeLa shCtrl cells after 72 h of growth in 0 mM glucose MEM media with or without 0.4 mM serine and 0.4 mM glycine (Ser/Gly). Cells were treated with PEPCK-M inhibitor iPEPCK-2 (5 μM). Fold change was calculated to minus Ser/Gly condition. Significance was determined using two-way Anova with Sidak multiple comparison post-test analysis. **Supplementary Figure 5.** Glucose content and PEPCK-M activity modulates Proline metabolism. **(A)** Quantitative real time PCR analysis of PYCR1 and PRODH/POX mRNA expression levels in HeLa cells grown in glucose exhaustion (1 mM) conditions for 24 h. Values were normalized to high glucose (25 mM) condition which is represented by dotted line. Statistical significance to shCtrl was determined by using unpaired two-tailed Student's *t*-test. **(B)** ^13^C enrichment of proline in HeLa cells were exposed to 2 mM [U-^13^C]glutamine for 4 h in the DMEM media containing 10% dFCS and 25 mM glucose. Incorporation of ^13^C was analyzed using GC-MS. One-way Anova with Sidak multiple comparison post-test analysis did not show significant differences. **(C)** Proline effects (5 mM) on viability after iPEPCK-2 treatment (4.3 μM) on HCT116 colon carcinoma cells grown in DMEM media lacking glucose for 48 h. Cell viability was measured using an MTT assay Statistical significance was determined by using unpaired two-tailed Student's *t*-test. **Supplementary Figure 6.** Loss of PEPCK-M silencing in xenografts from HeLa cell clones. (A) Tumor growth of HeLa cells implanted into mammary fat pad of BALB/C nude mice. One-way Anova with Sidak multiple comparison post-test analysis did not show significant differences. (B) Western blot analysis of PEPCK-M in tumors from **A**. Gamma tubulin was used as loading control.

## Data Availability

No datasets were generated in the course of the work presented here; therefore, data archival does not apply for this study. All data is available from the authors and will be archived for a certain amount of time subject to regulations by the institution (University of Barcelona).
