# Supplementary material for: PEPCK-M recoups tumor cell anabolic potential in a PKC-ζ-dependent manner
Source: Cancer Metab. 2021 Jan 7;9:1. doi: 10.1186/s40170-020-00236-3 (PMC7791766; doi:10.1186/s40170-020-00236-3)
Supplement: Supplementary file 1 — Additional file 1: Supplementary Table 1. Oncomine datasets reference list. Log2 median centered ratio of PCK1 and PCK2 in different cancers analyzed with Oncomine (https://www.oncomine.org; Rhodes et al., 2004). Analysis type: Cancer versus Normal, threshold at p=1E-5 and top gene rank 10%. Missing values for PCK1 expression were not significant or were not analyzed in given dataset. Reference to original study is listed. [file 40170_2020_236_MOESM1_ESM.pdf]

Supplementary Table 1: Oncomine datasets reference list. Log2 median centered ratio of *PCK1* and *PCK2* in different cancers analyzed with Oncomine (https://www.oncomine.org; Rhodes *et al.*, 2004). Analysis type: Cancer versus Normal, threshold at p=1E-5 and top gene rank 10%. Missing values for PCK1 expression were not significant or were not analyzed in given dataset. Reference to original study is listed.

Tissue of origin	Log2 median centered ratio		Oncomine dataset (reference)
	PCK2	*PCK1*	
Bladder	1.864	-	Sanchez-Carbayo Bladder 2 (Sanchez-Carbayo *et al*, 2006)
Bladder	1.927	-	Dyrskjot Bladder 3 (Dyrskjøt *et al*, 2004)
Bladder	2.101	-	Dyrskjot Bladder 3 (Dyrskjøt *et al*, 2004)
Bladder	3.063	-	Sanchez-Carbayo Bladder 2 (Sanchez-Carbayo *et al*, 2006)
Blood	2.098	-	Pyeon Multi-cancer (Pyeon *et al*, 2007)
Blood	1.501	-	Zhan Myeloma 3 (Zhan *et al*, 2008)
Blood	1.542	-	Zhan Myeloma 3 (Zhan *et al*, 2008)
Blood	1.914	-	Brune Lymphoma (Brune *et al*, 2008)
Blood	2.097	-	Brune Lymphoma (Brune *et al*, 2008)
Blood	2.641	-	Piccaluga Lymphoma (Piccaluga *et al*, 2007)
Blood	4.610	-	Andersson Leukemia (Andersson *et al*, 2007)
Blood	5.489	-	Andersson Leukemia (Andersson *et al*, 2007)
Blood	5.602	-	Piccaluga Lymphoma (Piccaluga *et al*, 2007)
Blood	-3.378	-8.432	Finak Breast (Finak *et al*, 2008)
Breast	1.572	-2.608	Curtis Breast (Curtis *et al*, 2012)
Breast	1.618	-1.949	Curtis Breast (Curtis *et al*, 2012)
Breast	1.636	-	Curtis Breast (Curtis *et al*, 2012)
Breast	1.685	-1.893	Curtis Breast (Curtis *et al*, 2012)
Breast	1.704	-1.98	Curtis Breast (Curtis *et al*, 2012)
Breast	1.747	-1.971	Curtis Breast (Curtis *et al*, 2012)
Breast	1.809	-1.982	Curtis Breast (Curtis *et al*, 2012)
Breast	1.884	-	Ma Breast 4 (Ma *et al*, 2009)
Breast	2.077	-2.043	Curtis Breast (Curtis *et al*, 2012)
Breast	1.572	-	Pyeon Multi-cancer (Pyeon *et al*, 2007)
FRS	2.333	-	Biewenga Cervix (Biewenga *et al*, 2008)
FRS	3.470	-	Yoshihara Ovarian (Yoshihara *et al*, 2009)
Gastrointestinal	-4.035	-	Cho Gastric (Cho *et al*, 2011)
Gastrointestinal	-2.409	-	Chen Gastric (Chen *et al*, 2003)
Gastrointestinal	-2.162	-	Skrzypczak Colorectal 2 (Skrzypczak *et al*, 2010)
Gastrointestinal	-2.020	-	Kaiser Colon (Kaiser *et al*, 2007)

Gastrointestinal	-1.741	-	Kaiser Colon (Kaiser *et al*, 2007)
Gastrointestinal	-1.736	-	Kaiser Colon (Kaiser *et al*, 2007)
Gastrointestinal	-1.730	-11.78	Gaedcke Colorectal (Gaedcke *et al*, 2010)
Gastrointestinal	-1.647	-	Kaiser Colon (Kaiser *et al*, 2007)
Gastrointestinal	-1.576	-10.11	Skrzypczak Colorectal 2 (Skrzypczak *et al*, 2010)
Gastrointestinal	-1.546	-6.755	Skrzypczak Colorectal 2 (Skrzypczak *et al*, 2010)
Gastrointestinal	-1.501	-5.458	Kaiser Colon (Kaiser *et al*, 2007)
Heptocellular	-2.862	-5.15	Chen Liver (Zhou *et al*, 2002)
Heptocellular	-2.862	-10.35	Roessler Liver (Roessler *et al*, 2010)
Hepatocellular	-3.400	-7.603	Roessler Liver (Roessler *et al*, 2010)
Liposarcoma	-3.191	-	Barretina Sarcoma (Barretina *et al*, 2010)
Liposarcoma	-2.817	-	Barretina Sarcoma (Barretina *et al*, 2010)
Renal	-10.41	-14.78	Beroukhim Renal (Beroukhim *et al*, 2009)
Renal	-9.677	-23.35	Beroukhim Renal (Beroukhim *et al*, 2009)
Renal	-8.552	-	Lenburg Renal (Lenburg *et al*, 2003)
Renal	-7.623	-	Gumz Renal (Gumz *et al*, 2007)
Renal	-4.330	-	Jones Renal (Jones *et al*, 2005)
Renal	-3.547	-8.938	Jones Renal (Jones *et al*, 2005)
Renal	-3.474	-18.9	Jones Renal (Jones *et al*, 2005)
