# Supplementary material for: PEPCK-M recoups tumor cell anabolic potential in a PKC-ζ-dependent manner
Source: Cancer Metab. 2021 Jan 7;9:1. doi: 10.1186/s40170-020-00236-3 (PMC7791766; doi:10.1186/s40170-020-00236-3)

Supplementary Fig 1

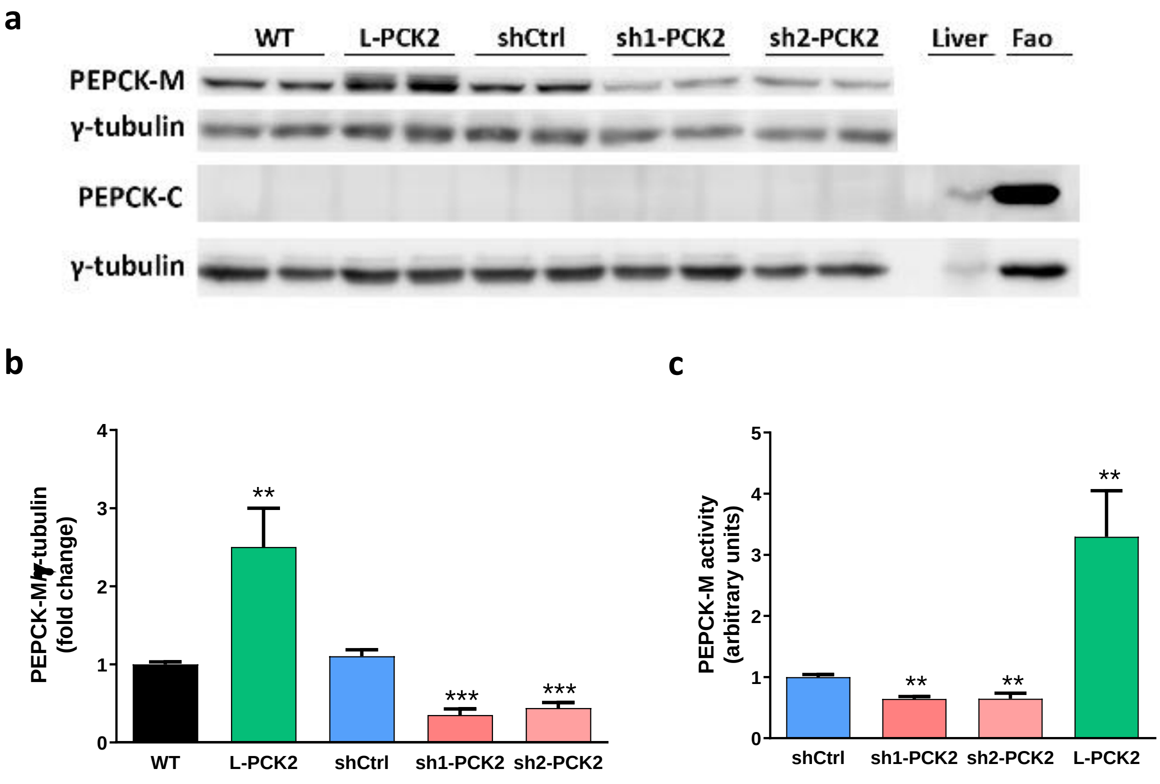

Supplementary Fig 2

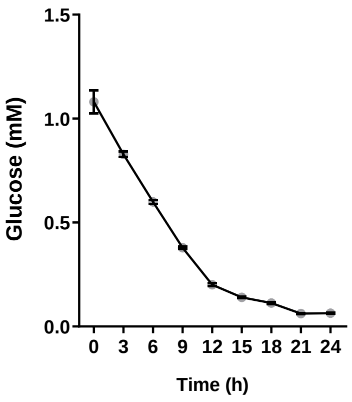

Supplementary Fig 3

a

Gly	shCtrl	sh1-PCK2	L-PCK2
m+0	99.91	99.89	99.97
m+1	0.07	0.09	0.03
m+2	0.02	0.02	0.00

Ser	shCtrl	sh1-PCK2	L-PCK2
m+0	100.00	100.00	99.99
m+1	0.00	0.00	0.01
m+2	0.00	0.00	0.00
m+3	0.00	0.00	0.00

b

Gly	HG DMEM	MEM
m+0	99.98	100.00
m+1	0.02	0.00
m+2	0.00	0.00

Ser	HG DMEM	MEM
m+0	99.90	100.00
m+1	0.00	0.00
m+2	0.00	0.00
m+3	0.10	0.00

Supplementary Fig 4

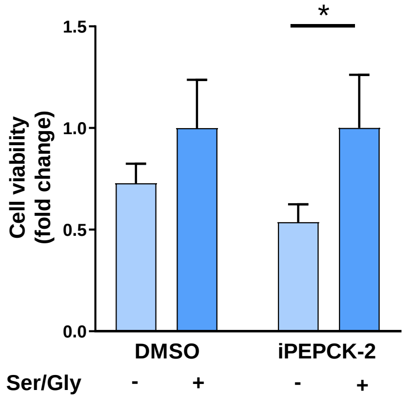

Supplementary Fig 5

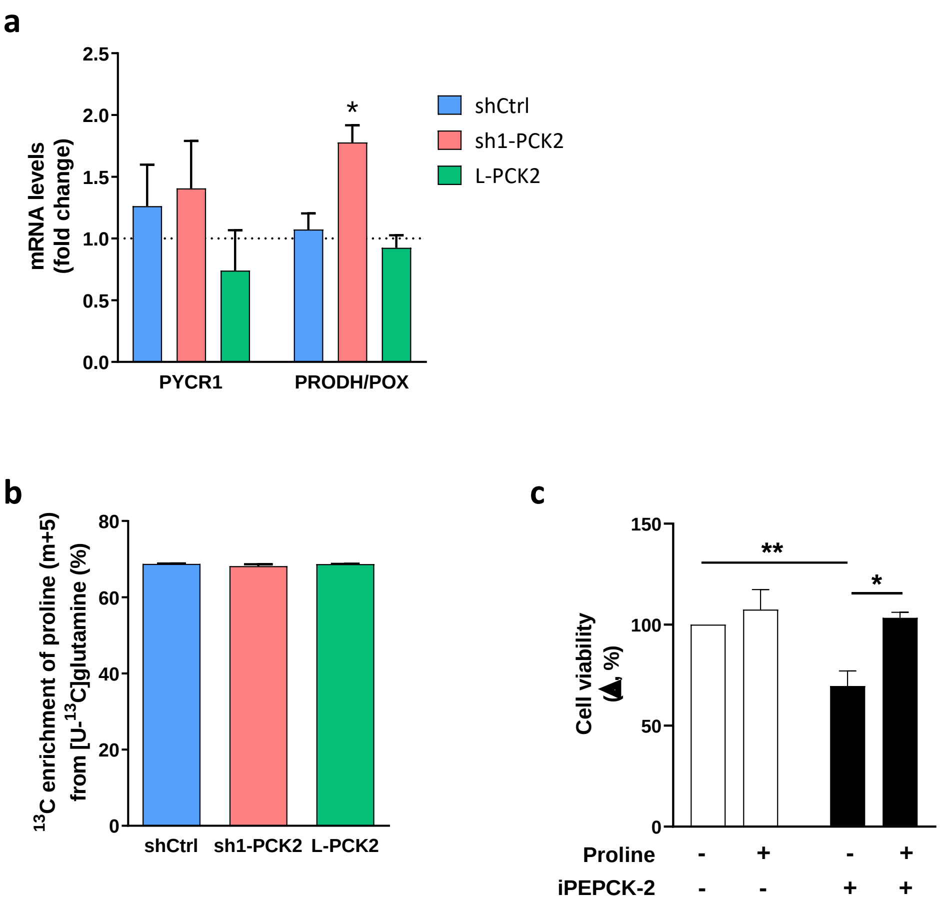

Supplementary Fig 6

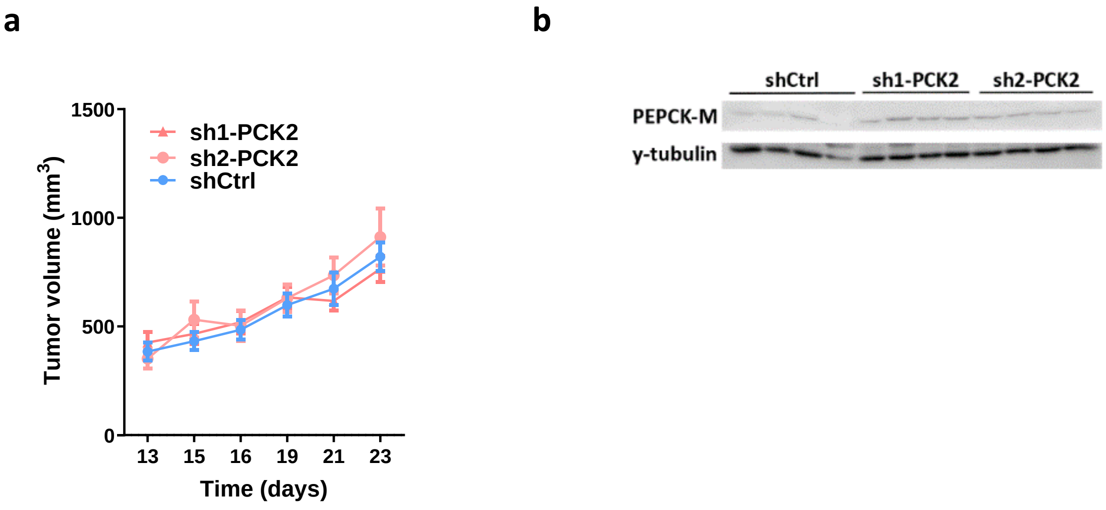

Supplement: Supplementary file 2 — Additional file 2: Supplementary Figure 1. HeLa model with stable silencing and overexpression of PEPCK-M. Silencing and overexpression were obtained using lentiviral vectors containing inserts of shRNA against PCK2 or inserts of PCK2 cDNA, respectively. (A) Western blot analysis of mitochondrial and cytosolic PEPCK expression levels in HeLa cells with altered PEPCK-M expression levels: overexpressed (L-PCK2), basal (shCtrl and WT) and knocked down PEPCK-M (sh1-PCK2 and sh2-PCK2). As a positive control of PEPCK-C expression, extracts from mouse liver and Fao hepatoma cells were used. (B) Western blot quantification of PEPCK-M protein abundance in HeLa modified lines. PEPCK-M expression was normalized by gamma tubulin. Results are represented as fold change to HeLa WT. One-way Anova with Sidak multiple comparison post-test analysis indicate significance versus WT. (C) PEPCK-M enzymatic activity in HeLa shCtrl, sh1-PCK2, sh2-PCK2 and L-PCK2 cells grown in basal conditions was measured by production of NADH. One-way Anova with Sidak multiple comparison post-test analysis indicate significance versus shCtrl. Supplementary Figure 2. Time course of glucose depletion under glucose exhaustion conditions. HeLa shCtrl cells were washed 3 times with PBS and treated with DMEM medium containing 1 mM glucose. Concentration of glucose in medium was measured every 3 h, up to 24 h. Supplementary Figure 3. 13C enrichment of serine and glycine in HeLa cells. (A) Cells were exposed to 2 mM [U-13C]glutamine for 4 h in the DMEM media containing 10% dFCS and 25 mM glucose. Incorporation of 13C into proline was analyzed using GC-MS. (B) HeLa shCtrl cells were exposed to 2 mM [U-13C]glutamine for 4 h in the DMEM media containing 10% dFCS and 25 mM glucose or media lacking serine and glycine (MEM) containing 10% dFCS and 5 mM glucose. Incorporation of 13C was analyzed using GC-MS. Negative values were set as 0. Supplementary Figure 4. PEPCK-M inhibition with iPEPCK-2 effects on viability are [file 40170_2020_236_MOESM2_ESM.pdf]
